# Thermal Resilience of Feeding Kinematics May Contribute to the Spread of Invasive Fishes in Light of Climate Change

**DOI:** 10.3390/biology5040046

**Published:** 2016-11-25

**Authors:** Ralph Turingan, Tyler Sloan

**Affiliations:** Department of Biological Sciences, Florida Institute of Technology, 150 West University Boulevard, Melbourne, FL 32901, USA; turingan@fit.edu

**Keywords:** global warming, prey-capture, suction feeding, organismal performance, thermal tolerance

## Abstract

As a consequence of global warming, tropical invasive species are expected to expand their range pole-ward, extending their negative impacts to previously undisturbed, high-latitude ecosystems. Investigating the physiological responses of invasive species to environmental temperature is important because the coupled effects of climate change and species invasion on ecosystems could be more alarming than the effects of each phenomenon independently. Especially in poikilotherms, the rate of motion in muscle-driven biomechanical systems is expected to double for every 10 °C increase in temperature. In this study, we address the question, “How does temperature affect the speed of jaw-movement during prey-capture in invasive fishes?” Kinematic analysis of invasive-fish prey-capture behavior revealed that (1) movement velocities of key components of the feeding mechanism did not double as water temperature increased from 20 °C to 30 °C; and (2) thermal sensitivity (Q10 values) for gape, hyoid, lower-jaw rotation, and cranial rotation velocities at 20 °C and 30 °C ranged from 0.56 to 1.44 in all three species. With the exception of lower-jaw rotation, Q10 values were significantly less than the expected Q10 = 2.0, indicating that feeding kinematics remains consistent despite the change in environmental temperature. It is conceivable that the ability to maintain peak performance at different temperatures helps facilitate the spread of invasive fishes globally.

## 1. Introduction

Projected variations in global temperature as a consequence of climate change have underscored renewed interest in addressing questions about temperature-dependent organismal performance [1,2,3,4,5]. The re-emergence of emphasis on ecological physiology of organisms is rooted in the notion that physiological traits are strong determinants of species response to climate change [6,7,8,9,10,11,12,13]. In light of the climate change phenomenon, what mechanisms underlie organismal response to environmental temperature variations? In an attempt to establish the foundation for continuing investigations addressing this central question, this study was designed to explore the effects of temperature on whole-organism performance, specifically prey-capture kinematics, in three orders of invasive-teleost fishes. To our knowledge, these are the first three invasive species used to investigate the effects of environmental temperature on feeding kinematics in light of climate change. The use of invasive fishes to examine temperature-dependent performance increases the relevance of this study because of the belief that the coupled effects of climate change and species invasion on ecosystems are more alarming than the effects of each taken independently [14]. It is predicted that, as a consequence of global warming, tropical invasive species expand their range poleward, thus, extending their well-known negative biological impacts to previously undisturbed, high-latitude ecosystems [14,15,16].

The effects of environmental temperature on physiological processes are widespread in both animals and plants, but in fishes its effects on skeletal-muscle performance and property have been the focus of most basic and applied physiological studies. It is expected that the direct effects of environmental temperature on body temperature mediate physiological processes, most especially metabolic rate. In fishes, as with any poikilothermic vertebrate, physiological performance peaks at a narrow range of body temperature, and environmental temperature remains a major constraint in their range of distribution [4,5,17,18,19,20]. Metabolic rate, in general, and contraction rate of skeletal muscle fibers, in particular, increase with environmental temperature until a threshold level is achieved, after which these rates decline with further increase in environmental temperature [18,21,22,23,24,25,26]. The ability of an individual to perform a certain task (e.g., prey capture) driven by a temperature-dependent process (e.g., rate of contraction of buccal-expansion muscles) is constrained by the reduction in biological rates as a consequence of decrease in environmental temperature. Furthermore, the expression of temperature-induced changes in fish-muscle physiology varies according to different temporal scales (i.e., daily, seasonal, or life-history, developmental time scales) and levels of organization (i.e., from molecular to organismal to ecosystem) [27]. For example, intracellular and extracellular ionic concentration and acid balance are destabilized by instant changes in environmental temperature [28]. Seasonal temperature change may induce modifications of the properties and composition of the contractile elements of the muscle fiber [27]. This time scale may allow fish to acclimate to the new ambient temperature and drive plastic response in muscle-fiber phenotypes and trigger behavioral and other mechanisms that buffer temperature effects and maintain homeostasis [28,29,30]. At the whole-organism level, responses to environmental temperature change may be taxon- and ontogenetic- specific. Indeed, environmental temperature has a profound influence on the fish’s ability to successfully accomplish relevant tasks such as swimming, feeding, mating, and escaping from predators.

Interestingly, previous studies investigating temperature effects on whole-organism performance in fishes have revealed mixed results. The kinematics of routine-swimming in teleost fishes have been largely consistent with physiological expectations. For example, swimming velocity doubles in response to a 10 °C increase in environmental temperature, that is, Q10 values are at least 2.0 [31,32,33,34]. The physiological quotient (Q10 value) indicates the magnitude of change in biological rates (e.g., swimming speed) for every 10 °C change in temperature [31,32,33,34]. An attempt to arrive at a consensus on the effects of temperature on feeding kinematics in teleost fishes remains unsuccessful perhaps because of the differences in the experimental design employed by the relatively few studies investigating this subject matter. In investigating how decrease or increase in environmental temperature, between 18 °C and 24 °C, affect prey-capture performance, Wintzer and Motta [19] concluded that it took bluegill (*Lepomis macrochirus*) longer to achieve maximum gape and lower-jaw rotation as water temperature decreased. DeVries and Wainwright [20] found that a 15 °C decrease in temperature caused only the time to reach maximum gape, among all timing parameters that underlie suction feeding performance in largemouth bass (*Micropterus salmoides*), to slightly increase. In Sloan and Turingan [35], and Turingan and Sloan [36], Repeated Measures Multivariate Analysis of Covariance revealed that environmental temperature, raised from 20 °C to 30 °C at a rate of 1 °C daily, had no effect on the magnitude and timing of prey-capture kinematics of nonnative teleost fishes in Florida, USA. Considering that the ability to successfully capture prey determines individual survivorship, it is imperative to elucidate how environmental temperature mediates prey-capture performance in organisms.

Three invasive Florida fishes—Pike killifish, *Belonesox belizanus* (Cyprinidontiformes); lionfish, *Pterois volitans* (Scorpaeniformes); and Mayan cichlid, *Cichlasoma urophthalmus* (Perciformes)—were used to determine how variable or consistent whole-organism response is, particularly in invasive species, to environmental temperature. Consistent with the prediction of climate-driven range expansion of invasive-species poleward, these invasive fishes have continued to extend their distribution northward from where they were introduced in south Florida [37,38,39,40]. The average annual temperature within the current distribution of these invasive fishes ranges between 20 °C and 30 °C [41]. This is used as the basis for the selection of experimental temperatures in this study. 

Identified as one of the most abundant nonnative fishes in Florida, *Belonesox belizanus* is native to waters (temperature is 25–37 °C) in Mexico and Central America, and was introduced into a ditch in south Florida in the late 1950s [37,38,39,40,41,42,43]. It is a specialist predator, feeding on small fishes using a feeding mechanism that is well designed for piscivory. This piscivore is capable of achieving a large gape with its elongated premaxillae and mandibles lined with large teeth [44,45,46]. The ability of pike killifish to independently rotate its premaxilla posterodorsally, facilitated by the premaxillomandibular ligament and a twisting maxilla, further enhances gape formation [45].

*Pterois volitans* is native to the Indo-Pacific Ocean (temperature is 22–28 °C) [47]. After its initial introduction in south Florida in the early 1990s, it has rapidly expanded its invasive population southward to the Caribbean and northward along the Atlantic coast of the USA [48,49,50,51]. The invasive lionfish has been identified as the likely worst threat to marine biodiversity in the Mid-Atlantic, Gulf of Mexico, and Caribbean regions [48,49,50,51]. This invasive predator feeds on a diverse group of fishes in the region, including 21 families and 41 species of teleost fishes, the majority of which have commercial, recreational, and ecological importance [50,52]. The predatory success of the lionfish is perhaps enhanced by its ability to modulate its suction-feeding repertoire, including a characteristic rapid-strike on more mobile, elusive fish and crustacean prey [53].

The native distribution of *Cichlasoma urophthalmus* ranges from eastern Mexico to Nicaragua (temperature is 22–39 °C) [54]. Following its introduction into south Florida in the 1980s, it has spread into north Florida and the Florida Bay regions [55,56,57]. Perhaps among the traits that enable the invasive Mayan cichlid to spread northward in Florida is its tolerance to extreme variations in salinity [55,56,57,58] and temperature [56,58]. In addition, the invasive Mayan cichlid has a generalist diet, feeding on detritus, plants, invertebrates and fish [59,60,61,62]. Its feeding apparatus includes an oral-jaw mechanism for prey capture and a well-developed pharyngeal-jaw apparatus for prey-processing [63].

This study was designed to test the hypothesis that the velocity of prey-capture kinematics, particularly buccal expansion and compression behaviors powered by skeletal-muscle in teleost fishes doubles when ambient-water temperature is raised by 10 °C.

## 2. Materials and Methods

**Figure 1:**
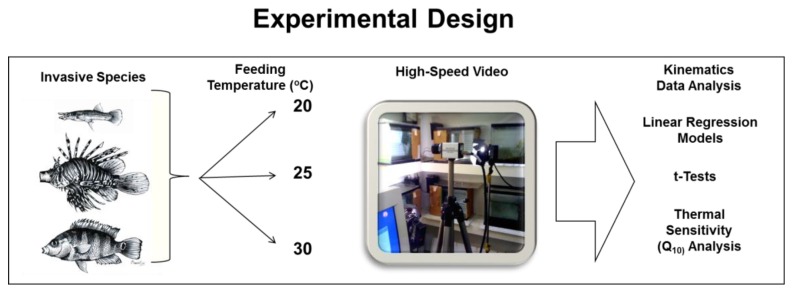
Diagram depicting the experimental design investigating the effects of environmental temperature on the kinematic velocity to reach maximum gape, hyoid depression, cranial rotation, and lower-jaw rotation. Four individuals of each invasive species *Belonesox belizanus*, *Pterois volitans*, and *Cichlasoma urophthalmus* were filmed while feeding on live-fish prey at 20 °C, 25 °C, and 30 °C using high-speed video. The best four films of each individual feeding at each temperature were digitized to measure the four kinematic velocities stated above and to calculate Q10 values. Kinematic velocities and Q10 values were subjected to the appropriate statistical tests to determine the effects of temperature on prey-capture performance.

Four *B. belizanus*, collected from the Florida Everglades National Park, four *C. urophthalmus*, collected from Merritt Island, Florida, and four *P. volitans*, collected from Port St. Lucie, Florida were acclimated to 20 °C water temperature and trained for high-speed video in the fish ecophysiology laboratory at Florida Institute of Technology for two weeks before the experiment was initiated. Each fish was housed in 38 L filming tanks filled with water that matched their Florida habitats: 0 ppt for *B. belizanus*; 24 ppt for *C. urophthalmus*, and 35 ppt for *P. volitans*. The twelve fishes were subjected to a repeated measures experimental design, in which, each fish was filmed successively in each of the three experimental temperatures 20 °C, 25 °C and 30 °C (Figure 1). Temperature in each filming tank was raised from 20 °C to the higher filming temperatures at a rate of 1 °C daily using a water heater. Once the experimental temperature was achieved, feeding sessions were recorded every other day from each fish. Each fish was filmed using a RedLake High-Speed Motionscope 2000S camera with a shutter speed of 1/1000 s at 250 frames per second while feeding on live mosquitofish (*Gambusia holbrooki*) prey at 20 °C, 25 °C and 30 °C. Prey was maintained at 20 °C ambient-room temperature. Prey girth was about 80% of peak gape of each fish; previous analyses of feeding kinematics in these fishes indicated that this relative prey-size elicited maximum prey-capture performance in fish [35,36,46]. The effects of temperature on the prey was not investigated in this study. Experimental fish was not fed 1–2 days before each recording session to ensure fish was motivated to eat and exhibit maximum performance during feeding trials [19,20].

**Figure 2:**
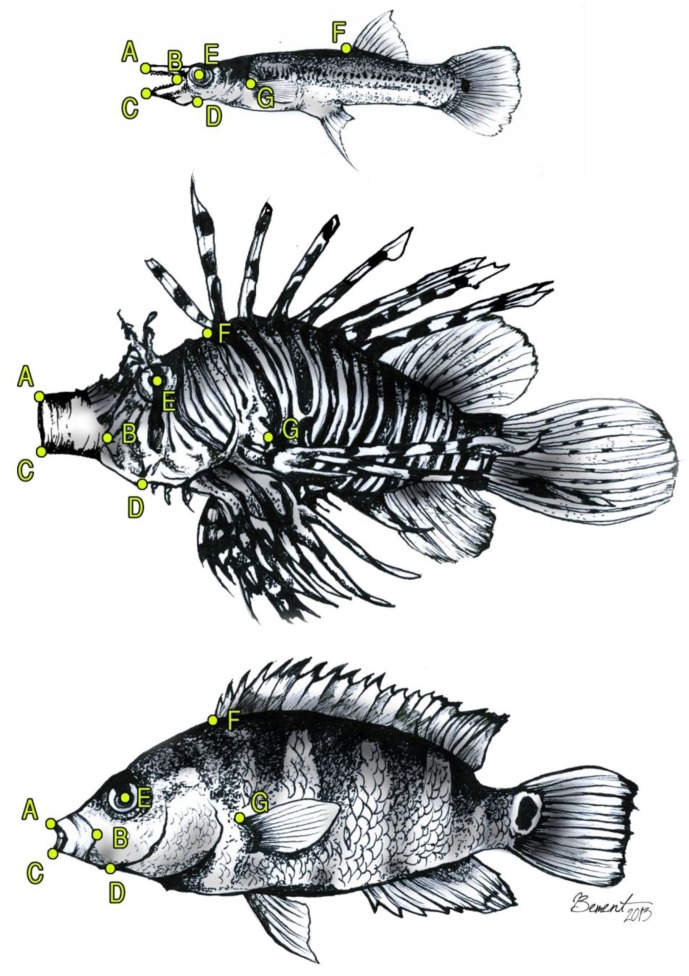
Diagram of the pike killifish, *Belonesox belizanus* (**top**), lionfish, *Pterois volitans* (**middle**), and Mayan cichlid, *Cichlasoma urophthalmus* (**bottom**) showing the homologous hotspots used to measure peak gape (= maximum distance measured from the anteriormost tip of the premaxilla (A) to the anteriormost tip of the dentary (C)), peak hyoid depression (= maximum distance between the center of the eye (E) to the anteriormost tip of the hyoid bar (D)), peak lower-jaw rotation (= maximum posteroventral rotation of the lower-jaw, measured as the angle formed by line segments AB (= jaw-joint) to BC), and peak cranial rotation (= maximum posterodorsal rotation of the neurocranium, measured by the angle formed by line segments AG (= dorsal tip of the pectoral-fin base) to GF (= anterior tip of the dorsal-fin base)).

Each fish was filmed until at least 10 feeding bouts were recorded in which the fish was perpendicular to the camera and exhibited maximum prey-capture performance. The best four films were analyzed per fish at each of the three experimental temperatures using MaxTRAQ (Version 2.2.4.1 Innovision Systems, Inc., Columbiaville, MI, USA). Each film was played back frame-by-frame to measure maximum gape (mm), hyoid depression (mm), lower-jaw rotation (degree), and cranial rotation (degree), as well as the time (ms) to reach each of these maximum kinematic-displacement variables. Time to reach each of these maximum kinematic-displacement variables was calculated using the frame prior to mouth opening as time = 0. Reference points (i.e., kinematic hotspots) used to measure these variables are illustrated in Figure 2. Average velocity was calculated as the value of the maximum kinematic-displacement variable divided by the corresponding time to reach this maximum displacement: Gape Velocity = Maximum Gape ÷ Time to Reach Maximum Gape; Hyoid Velocity = Maximum Hyoid Depression ÷ Time to Reach Maximum Hyoid Depression; Lower-Jaw Rotation Velocity = Maximum Lower-Jaw Rotation ÷ Time to Reach Maximum Lower-Jaw Rotation; Cranial Rotation Velocity = Maximum Cranial Rotation ÷ Time to Reach Maximum Cranial Rotation. Physiological quotient (Q_10_) was calculated for each of the kinematic-velocity variables as Q10 = (Kinematic Velocity at 30 °C/Kinematic Velocity 20 °C) (modified from Schmidt-Nielsen [64]).

Each of the four kinematic-velocity variables was subjected to a Model I least-squares regression against temperature to define the model y = a + bx; where y = kinematic velocity, a = intercept, b = slope, and x = environmental temperature (=20 °C, 25 °C, and 30 °C). To test the hypothesis that the Q10 of each of the kinematic-velocity variables was different from 2.0, a series of Paired t-Tests were conducted to compare the empirical Q10 values with the theoretical Q10 value of 2.0. All statistical tests were conducted using R.

After the experiment, each fish was sacrificed using an overdose of MS-222 solution, fixed in 10% formalin solution, and then stored in 75% ethanol solution. All specimens have been stored appropriately in the fish ecophysiology and evolution laboratory at Florida Institute of Technology for use in current and future teaching and research. All procedures for housing, maintaining and sacrificing experimental fishes strictly followed the guidelines and procedures of the Institutional Animal Care and Use Committee (IACUC) of the Florida Institute of Technology (IACUC Approval # 101202).

## 3. Results

**Figure 3:**
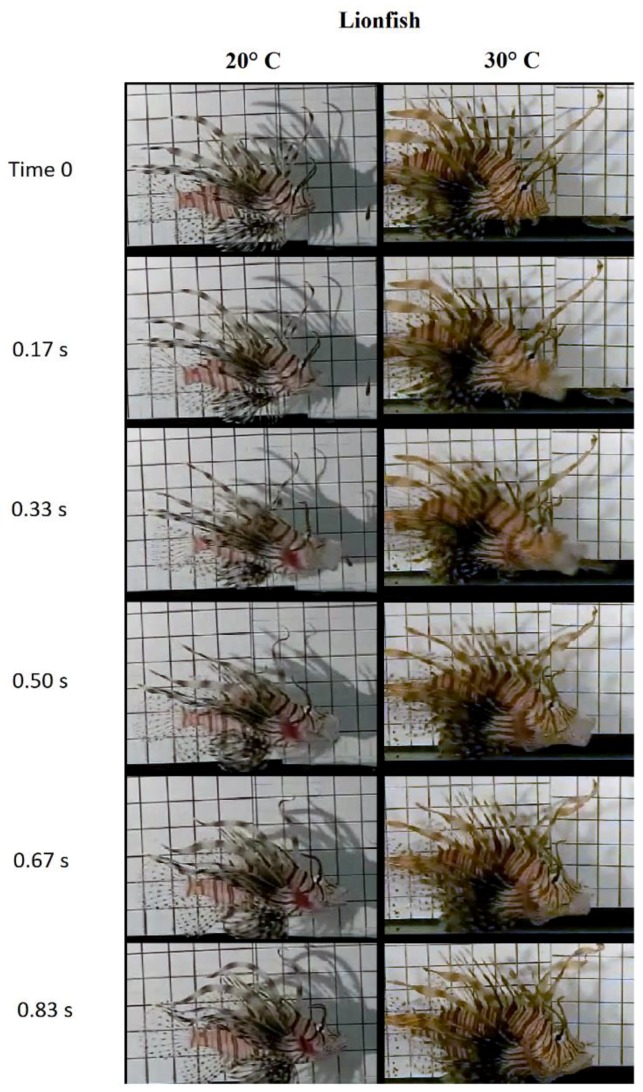
Select frames of representative films of two lionfish showing the sequence of kinematic events during prey capture in lionfish, *Pterois volitans*, at 20 °C, and 30 °C. Note that all species of invasive fishes successfully captured prey in both temperatures.

The three Florida invasive-fish species fed voraciously upon introduction of the prey during feeding-recording sessions at each of the three environmental temperatures, 20 °C, 25 °C, and 30 °C (Figure 3). Examination of the films revealed that the feeding behavior of each fish was consistent with previous studies [35,36,46,53]. The pike killifish stalked its prey and within a very short distance between the predator and the prey, the fish lunged toward the prey, opened its mouth widely and snapped at the prey. The lionfish used its pectoral fins to herd the prey closer to its mouth before suction feeding to capture prey. The Mayan cichlid behaved more aggressively; as soon as the prey was introduced into the filming tank, the cichlid rapidly swam toward the prey and suction-fed on it instantaneously.

**Figure 4:**
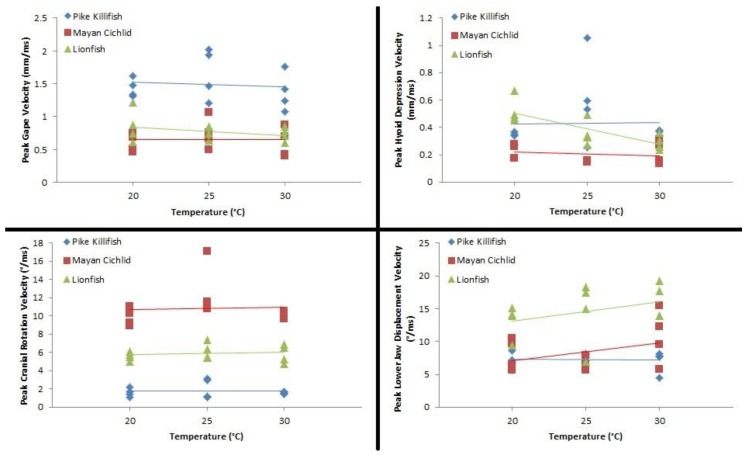
Scatterplot showing the relationship between each of the four kinematic-velocity variables and feeding temperature 20 °C, 25 °C, and 30 °C. Results of the regression analysis that quantified the effect of temperature on each of the kinematic events are presented in Table 1.

Linear regression models indicated that the average velocity of kinematic events during feeding in all three invasive species remained consistent across environmental temperatures, with the exception of the average velocity of hyoid depression in the Mayan cichlid and average velocity of lower-jaw rotation in the lionfish. Species-specific variation in elevation (=y-intercept, a) is apparent, but, the slopes of the regression, b, were not statistically different from zero, indicating that kinematic velocities were unaffected by environmental temperature (Table 1 and Figure 4).

**Figure 5:**
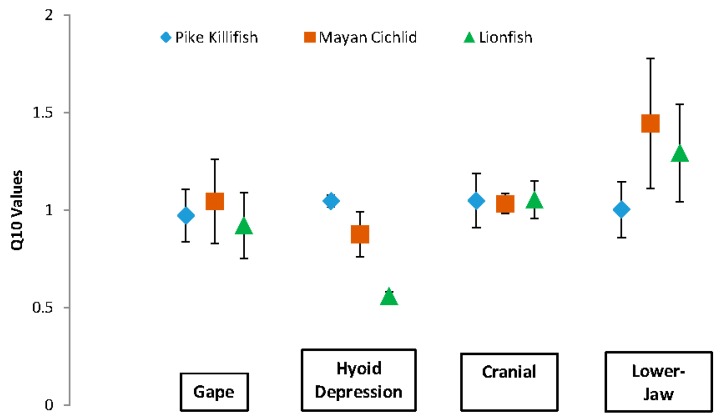
Mean Q10 values for kinematic-velocity in each of the three invasive-fish species. Note that except for the mean Q10 values for lower-jaw rotation in the Mayan cichlid and lionfish, all Q10 values of kinematic velocity were significantly less than the expected Q10 value of 2.0 for fish feeding at 20 °C and 30 °C. Error bars indicate standard error of the mean.

The mean Q10 values of each of the kinematic velocities were significantly less than the expected Q10 value of 2.0, with the exception of the average velocity of lower-jaw rotation in the lionfish and Mayan cichlid (Table 2 and Figure 5).

## 4. Discussion

Empirical evidence of how organismal performance is affected by environmental change advances our understanding of the consequences of climate change and invasion of nonnative species. Investigations into the combined effects of both phenomena on native-community structure and dynamics, as well as range expansion of invasive species, are especially important considering that their combined effects are perhaps more devastating than each taken independently ([64], www.invasivespecies.gov.). It has been predicted that as a consequence of the pole-ward warming of the earth, tropical-invasive species will continue to expand their range toward higher-latitude ecosystems at alarming rates [14,65]. Well known characteristics of invasive species that enable them to impart damage in the stability of native ecosystems include: (1) they have high propensity to introduce and spread diseases [66,67,68]; (2) they alter community and food web structure through competition and predation [38,69,70,71,72,73]; (3) they hybridize with native species [74,75,76]; and (4) they outcompete native species and ultimately displace and even drive native species to extinction [1,2,67,69]. The latter results in the reduction of biodiversity and may even lead to biological homogenization. Although empirical evidence demonstrating the direct and indirect interactions between invasive and native species, as well as the cause-effect mechanism underlying post-invasion changes in community structure of invaded ecosystems, are elusive, it is hypothesized that climate change exacerbates the negative impacts of invasive species to ecology and society [77,78]. In our attempt to contribute to the advancement of our understanding of the impacts of the invasive species and climate change coupling to ecosystem dynamics, our discussion of the results of this study centers on the question, “How do invasive fishes deal with variations in temperature within their invasive range?”

As poikilotherms, the feeding performance of invasive fishes are expected to conform with the known effects of temperature on the physiology and ecology of heterothermic, aquatic animals [79]. For example, first, the velocity of fin propulsion during swimming and mouth-opening during feeding, behaviors fueled by skeletal-muscle contraction and relaxation, are expected to double when ambient temperature is increased by 10 °C. This is because at the physiological level of analysis, there is a two-fold increase in the rate of muscular contraction and relaxation for every 10 °C increase in temperature (i.e., Q10 = 2.0) [18,21,22,23,24,31]. Second, at the ecological level of analysis, predictable seasonal cooling and warming of lakes and rivers have contributed to the evolution of acclimatization in teleost fishes [80,81,82]. Third, the food habit of some temperate fishes, such as largemouth bass, *Micropterus salmoides*, and pumpkinseed sunfish, *Lepomis gibbosus*, change seasonally, consistent with the seasonal cooling and warming of lakes or rivers in temperate ecosystems [80,81,82].

Results of this study, as well as those of Sloan and Turingan [35] and Turingan and Sloan [36] underscore the thermal independence of prey-capture performance in invasive-teleost fishes. Suction-feeding, which is the most dominant and generalized mode of prey-capture in teleost fishes, relies primarily on the high-speed movement of cranial elements such as the jaws, hyoid, suspensorium, and cranium during mouth opening and closing (see Figure 3; [46,83,84,85,86,87,88,89]). A successful strike and capture of prey relies heavily on the almost simultaneous and rapid expansion of the buccal cavity to generate subambient-pressure in the buccal chamber and mouth opening [83,84,85]. These kinematic events are accomplished primarily by the posterodorsal rotation of the cranium, depression of the hyoid apparatus, lateral extension of the suspensorial and opercular bones, and the posteroventral rotation of the lower-jaws [83,84,85,86,87,88,89]. These cranial movements are driven by skeletal muscles including the epaxialis, sternohyoideus, retractor arcus palatini, levator operculi, and dilator operculi [83,84,85,87]. It is well known that the rate of contraction of skeletal muscle is expected to at least double for every 10 °C increase in environmental and body temperature in ectotherms such as teleost fishes [20,31,33,34,90,91,92]. However, temperature has no significant effects on the velocity of movement of the key elements of the prey-capture mechanism in the three contrasting models of invasive species reported here. Although, on average, prey-capture kinematics significantly differ among the three invasive species, the absence of a temperature-induced change in kinematic velocity is evident in all three invasive species [35,36].

Thermal independence of fast-start behaviors and kinematics has been found in other vertebrate animals. Navas et al. [93] concluded that the Q10 for “jump take-off velocity and mean swimming velocity” in the frog *Rana temporaria* was lower than Q10 = 2.0. “Running velocity during burst activity” in several species of lizards were less affected by temperature, as evident in the low Q10 values for this behavior [94,95,96]. “Ballistic mouth opening and tongue projection dynamics” in toads (*Bufo terrestris*) were thermally independent [91]. Lack of temperature-induced variation in the dynamics of “ballistic mouth opening” was also evident in the frog *Rana pipiens* [96].

It is noteworthy that our results do not agree with the conclusions of the only two papers reporting the effects of temperature on the feeding kinematics of native teleost fishes. Prey-capture kinematics in North American native centrarchid fishes, bluegill *Lepomis microchirus*, and largemouth bass *Micropterus salmoides*, responded to environmental-temperature change in a manner that is consistent with physiological predictions [19,20]. Among other feeding-kinematic variables that were affected by temperature, it took longer for fishes to reach peak gape in colder than in warmer temperatures [19,20]. The prevalence of thermally independent prey-capture kinematics in invasive teleost fishes underscores the need to address how they compensate for the effects of temperature on the contractile properties and contraction velocity of skeletal muscles. A direct comparison between any of the invasive fishes in this study and an ecologically relevant (e.g., as a competitor or prey) native species in Florida is imperative, given the need to address the direct impacts of invasive on native species.

Translation of the physiological effects of temperature on muscle contractile properties to whole-animal performance may be mitigated by the central nervous system [27,97,98,99]. Central nervous-system governed compensatory mechanisms may allow whole animals to perform at optimum levels despite variation in environmental and body temperatures [100,101,102]. Such compensatory mechanisms likely include (1) plasticity in the recruitment of muscle fiber types [102,103,104,105]; (2) involvement of elastic strain energy storage and recovery in muscular and tendinous tissue [106,107]; (3) occurrence of temperature-induced change in acid-base balance in muscle fiber [107,108]; (4) plasticity of thermal sensitivity of myofibrillar ATPase activity [107,108]. For example, Rome et al. [104] concluded that at lower temperatures, carp (*Cyrprinus carpio*) recruited more fast-contracting (fast anaerobic) muscle fibers when environmental temperature was lower than ambient. Navas et al. [93] found that at 10 °C, optimal whole animal performance was accomplished by only 34% of muscle-power output. In the sartorius muscle of the toad *Bufo bufo*, Renaud and Stevens [107] demonstrated that short-term change in intracellular pH associated with decrease in water temperature from 25 °C to 5 °C was enough “to increase maximum force and, hence, power during isotonic shortening of muscle fiber, providing a short-term mechanism for compensation to low temperature”.

Global-climate change in general and global-temperature change in particular have important consequences for the performance of invasive species because of (1) the temperature-induced effects on physiological and mechanical processes [79,108]; (2) the likelihood that these physiological effects extend to whole-organism performance (e.g., [19,20]); and (3) the resilience of invasive species and the resistance of whole-organism performance to temperature change ([35,36]; this study]). These plausible avenues where the interplay between climate-change and invasive-species phenomena may be demonstrated need further investigation and confirmation.

## 5. Conclusions

The velocity of jaw movements during prey capture in the invasive fishes, *Belonesox belizanus, Pterois volitans*, and *Cichlasoma urophthalmus,* were statistically unaffected by water temperature. Within the range of temperature used in this study, all invasive fishes successfully captured their prey using a stereotypical suction-feeding kinematic pattern that is unaltered by temperature. It is plausible that this seemingly temperature-resilient behavior will facilitate the successful expansion of the invasive range of these tropical-fish species as a consequence of global warming.

## Figures and Tables

**Table 1:** Results of the regression analysis examining the effects of temperature on the kinematic velocities of maximum gape, hyoid depression, cranial rotation and lower-jaw rotation.

Kinematics	Species	a	b	*r^2^*	*p*
**Gape**	P	0.245	0.0343	0.0734	0.163
	L	1.797	−0.0357	0.0859	0.130
	M	1.821	−0.0376	0.0893	0.122
**Hyoid**	P	0.393	−1.14 × 10^−3^	4.53 × 10^−4^	0.914
**Depression**	L	0.738	−0.0162	0.0853	0.131
	M	0.980	−0.0267	0.227	0.010
**Cranial**	P	11.385	−0.243	0.0419	0.296
**Rotation**	L	8.808	−0.107	9.38 × 10^−3^	0.624
	M	−1.817	0.368	0.101	0.099
**Lower-Jaw**	P	13.365	−0.179	0.0286	0.390
**Rotation**	L	−3.568	0.610	0.233	0.009
	M	8.089	0.0664	3.61 × 10^−3^	0.761

Coefficients defining the regression equation, y = a + bx are shown. y = velocity to reach maximum Gape, Hyoid Depression, Cranial Rotation, and Lower-Jaw Rotation. a = intercept; b = slope. *R^2^* = Coefficient of Determination. p = probability associated with the null hypothesis that the slope of each regression is significantly different from zero. P = Pike killifish, *Belonesox belizanus*; L = Lionfish, *Pterois volitans*; M = Mayan cichlid, *Cichlasoma urophthalmus*.

**Table 2:** Results of the Paired *t*-Tests comparing the difference between mean Q10s of each of the kinematic velocity variables and the expected value of 2.0.

Kinematics	Species	Mean Q10	*t*-Statistic	Df	*p*
**Gape**	P	0.973	−7.6174	3	0.005
	L	0.922	−6.4448	3	0.008
	M	1.046	−4.3859	3	0.022
**Hyoid**	P	1.047	−30.0273	3	8.113 × 10^−5^
	L	0.559	−67.1036	3	7.239 × 10^−6^
	M	0.876	−9.8617	3	0.002
**Cranial**	P	1.049	−6.8275	3	0.006
**Rotation**	L	1.054	−9.7247	3	0.002
	M	1.033	−19.0769	3	3.145 × 10^−4^
**Lower-Jaw**	P	1.004	−6.9719	3	0.006
**Rotation**	L	1.294	−2.8106	3	0.063
	M	1.444	−1.6617	3	0.195

Relevant statistics are shown for P = Pike killifish, *Belonesox belizanus*; L = Lionfish, *Pterois volitans*; M = Mayan cichlid, *Cichlasoma urophthalmus*. Note that except for the Q10 of the velocity of lower-jaw rotation in Mayan cichlid and lionfish, all Q10 values of kinematic velocities are statistically less than the expected value of 2.0.
